# On the ^209^Po half-life error and its confirmation: an answer to the critique

**DOI:** 10.1007/s10967-015-4646-8

**Published:** 2015-12-08

**Authors:** Stefaan Pommé, Ljudmila Benedik

**Affiliations:** European Commission, Joint Research Centre, Institute for Reference Materials and Measurements, Retieseweg 111, 2440 Geel, Belgium; Jožef Stefan Institute, Jamova 39, 1000 Ljubljana, Slovenia

**Keywords:** Half-life, Polonium, Uncertainty, Tracer, Radioactivity, Environment

## Abstract

Pommé et al. published a paper claiming that the ^209^Po half-life is 20 % higher than the erroneous value of 102 (5) a used for 50 years. Collé and Collé published a critique saying that ‘this claim cannot withstand critical scrutiny’. In this work, counterarguments are presented to the critique. The experiment has been continued and a new intermediate half-life value of 122.7 (27) a was obtained. A brief review is made of the ^209^Po half-life value by Collé et al. and a recommended value of 122.9 (23) a is derived from both experiments.

## Introduction

The radionuclide ^209^Po is a widely used tracer for polonium in environmental and geophysical studies, and an accurate value of its half-life is required for unbiased decay corrections of the reference activity. In recent publications, Collé et al. [1, 2] provided evidence that the half-life of ^209^Po is roughly 25 % higher than the formerly recommended value of 102 (5) a [3]. From a data set of five massic alpha-particle emission rate measurements of a ^209^Po solution over a period of 20.7 years, Collé et al. derived a new ^209^Po half-life value of 125.2 (33) a [2].

In 2013, Pommé et al. [4] started continuous alpha emission measurements of two ^209^Po sources in a nearly-2*π* configuration on two 900 mm^2^ ion-implanted planar silicon detectors. Daily alpha energy spectra were taken in ideal, stable conditions: high resolving power of the alpha particles in the detectors, low noise and background, fixed geometrical configuration, high-purity source material, sufficient counting statistics, low dead time, unchanged electronics settings, etc. After 1 year, they published an intermediate result of these independent measurements, yielding a half-life of 120 (6) a [4], which supported the conclusions by Collé et al. [2].

In spite of the consistency of the results of Pommé et al. with those of Collé et al., Collé and Collé [5] recently published a report claiming that the work in Ref. [4] (referred to as the PSB paper) “*cannot withstand critical scrutiny*”. The aim of this paper is to provide answers to the points of critique raised by Collé and Collé [5], which will be referred to as the CC paper. In the “Reply to innuendo” section, comments are given on the insinuations and speculations which go beyond a mere factual scrutiny of the scientific evidence. In the “Reply to scientific arguments” section, the scientific arguments in the critique against the PSB work are refuted. In the “Comments on the *T*_1/2_(^209^Po) value by Collé et al.” section a brief review is made of the results of Collé et al. In the “Finding the best value” section an updated ^209^Po half-life value is presented.

## Reply to innuendo

### Relevance of the work

The PSB paper [4] was intended as a brief technical note conveying the intermediate half-life result after 1 year of measurement. The main message was that the obtained half-life value of 120 (6) a confirmed the invalidity of the old reference value. This was of great news value, considering that the decay data evaluation project (DDEP) reference value was at that time taken as the median value between two very discrepant data [6]. The confirmation of the new half-life value allowed for disposal of the old value of 102 (5) a as being discrepant.

The critique in the CC paper that “*Justification for the rush to publication is unclear.*” is contradictory to the level of importance that Collé et al. have attributed to their own, similar findings—“*a startling and remarkable revision*” [5]—in various publications and announcements listed in Ref. [5]. In an elegy by Journal of Physics G [7] marking this event, it was deservedly stated that “*An important aspect of this work is our belief that NIST is one of the few laboratories in the world with the unique attributes that could have detected this error and performed such decay measurements over a period of 20* *years. It results from decades of continuity in preserving precious radioactive materials, in having and maintaining well*-*documented standardization records, and in keeping an institutional memory defined by the collected set of facts, experiences, and know*-*how by a group of dedicated scientists.*”

Receiving recognition for having achieved a unique observation should not stand in the way of accepting equally compelling evidence from other laboratories obtained over a much shorter period in time.

### On the level of detail

Some of the critique pertains to the lack of detail provided to interpret the uncertainty components in the PSB paper. This remark is to some extent understandable, because the intention of the authors was (and still is) to continue the experiment over a period of several years and then to publish a detailed report with a final half-life result and extensive uncertainty analysis. The technical note was meant as a short paper in which the intermediate result and evidence were succinctly summarised. This intention was made very clear in the paper, as well as in correspondence with the reviewers and R. Collé. The conclusion of the PSB paper was nonetheless correct and the uncertainties in no way underestimated.

As recognised by Collé and Collé [5], it was the merit of Pommé to identify a problem with uncertainty propagation of medium and long-term instabilities on half-lives derived from decay curve measurements and to provide a convenient mathematical solution to it [8–10]. The critique that “*a fit tends to minimize the residuals and partly covers up the true medium*-*frequency effects*” and that “*uncertainties are greatly underestimated if one simply relies on goodness of the fit to the data*” is nothing more than an echo of the recommendations by Pommé on how to avoid underestimation of uncertainty in this type of measurement. Obviously, the authors are well aware of these problems and have applied good practice to the uncertainty estimation of the ^209^Po half-life.

The critics are too hasty to conclude that “*the author doesn’t heed his own advice*”. Their statement that “*half*-*life determinations, if made through the use of decay data,mustbe made over a sufficient time interval to adequately assess possible long*-*term influences*” certainly contains good advice, but is strictly speaking incorrect. There is no theoretical impediment to deriving a half-life value from a relatively short measurement campaign. The key is repeatability through high stability, which explains why the experiment by Pommé et al. is potentially better suited for half-life measurement of ^209^Po than the work of Collé et al. This point will be examined in more detail in this paper.

### On scientific debate and collaboration

The JRC and the JSI foster scientific debate and have a policy of openness. Therefore, the authors welcome the CC paper as an incentive to evaluate their own work (and that of Collé et al.) and to justify their decisions.

One of the curious items in the CC paper concerns their approach to the “*careful reanalysis of the original data*” (*sic*) of the PSB paper. Not only is the quality of their reanalysis questionable (see “The least-squares fit” section), they also did not use the original data. Instead, CC extracted an incomplete and approximate data set from a graph in the PSB paper by means of graphical-to-digital conversion software. Needless to say that this was an unnecessary complication, since they could have received the data from the authors on simple request.

CC criticise the DDEP evaluators [6] for using a median of the old value of 102 a and the half-life estimate of 128 a in Ref. [1], since “*it was not considered a new determination, because it was only based on two datum points over about 12* *years and that other possible effects ant its attendant components of variance were unknown*”. The DDEP evaluators compiled a ^209^Po half-life value of 115 (13) a, which was—in our opinion– at that time a reasonable decision given the availability of only those two discrepant data.

CC regret the fact that “*NIST scientists made several unsuccessful efforts to find willing collaborators to perform an absolute specific activity determination of the*^*209*^*Po half*-*life*”. Indeed, up to now the issue has not been readdressed with this technique, which is an important alternative to the decay curve approach [10]. This makes the PSB paper the only published experiment directly aimed at measuring the ^209^Po half-life, since the value by Collé et al. is the by-product of repeated standardisation work.

CC not only deny PSB the right to publish their (intermediate) result, they suspect “*a confirmation bias*” since the PSB value confirms the findings of Collé et al. They insinuate that PSB had seen the value in Ref. [2] and therefore dared to publish their result, which would not have happened if their value would have been 100 a or 140 a. CC speculate that PSB obtained a consistent result simply by “*mere fortuity*” (*sic*). The authors condemn these unjust allegations which question their scientific integrity.

Finally, CC also warn the reviewers and data compilers against a “*mere blind acceptance of any claimed measurement result and its associated uncertainty*” and plea for “*using judgement in evaluating the quality and substantiation of published results*”. The authors agree with this point of view, but come to an opposite conclusion as to which measurement provides the most trustworthy half-life value of ^209^Po.

## Reply to scientific arguments

### The least-squares fit

**Fig. 1:**
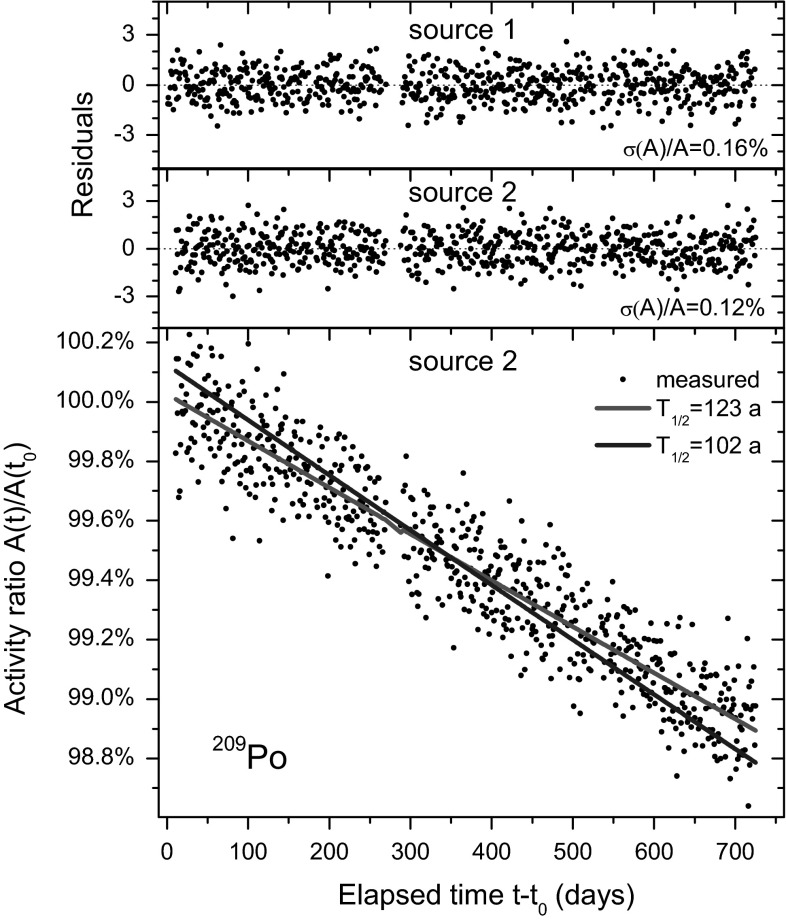
Decay curve of source 2 (*bottom*) and the fitted half-life of 123 a compared to the erroneous half-life of 102 a. Residuals to the fit of a 123 a decay curve for sources 1 and 2 (*top*), due to statistical variations in the decay rate

CC go at great length to demonstrate that the “best fit” to the PSB data is “*meaningless*” (*sic*). For the sake of argument, the measurement data of one of the sources in the PSB experiment have been updated to present day and are shown in Fig. 1. The graph shows the decay curve for source 2 with a fitted half-life of 123 a, as well as the residuals of the fit and the residuals of a similar fit to the data of source 1. Included in the bottom graph are fitted exponentials corresponding to a 102 a and 123 a half-life. The core of the lengthy critique by CC is that both curves fit the data equally well and that consistency of the 120 a fit result in the PSB paper with the result of Collé et al. is merely fortuitous. Admittedly, an intuitive gaze upon Fig. 1 may inspire some to draw such conclusions.

**Fig. 2:**
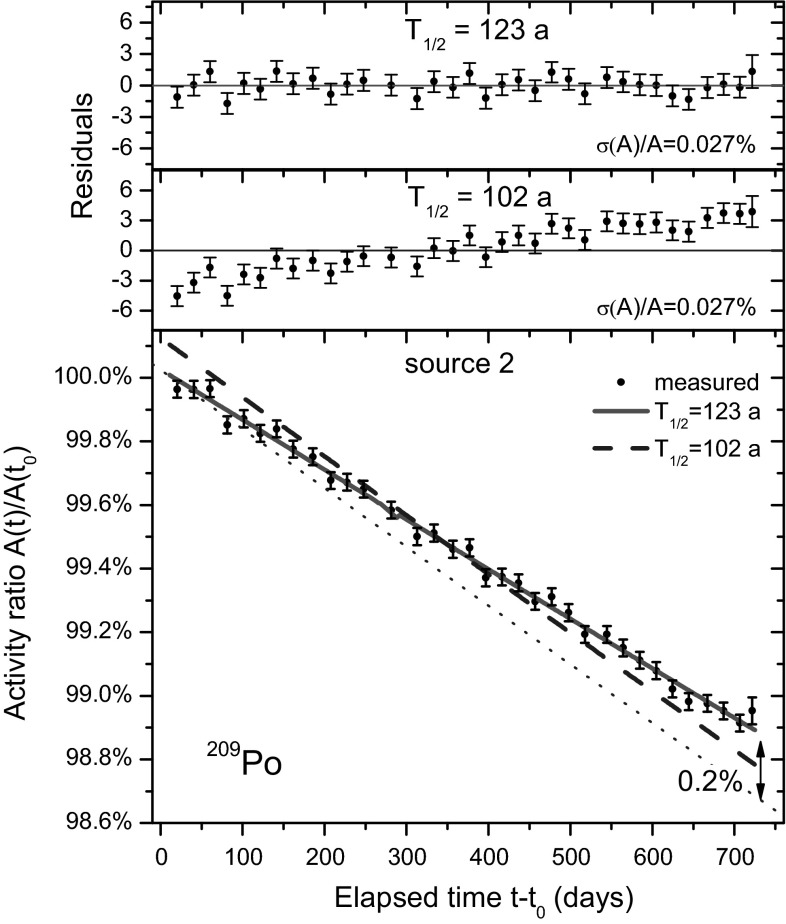
Same decay curve for source 2 as in Fig. 1, with average time, activity and residuals taken of groups of 20 data (*bottom*). Average residuals to an exponential function with a half-life value of 123 a and 102 a (*top*). The standard deviation of each data point is 0.027 %

**Fig. 3:**
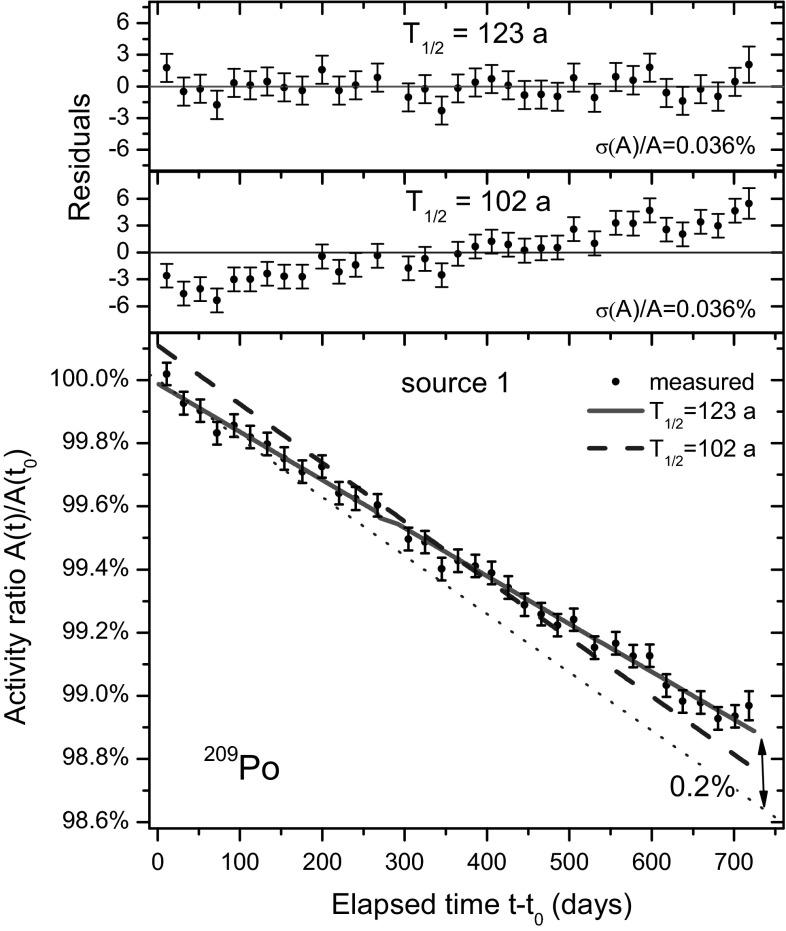
Same as in Fig. 2, for source 1. The standard deviation of each data point is 0.036 %

Nevertheless, this is a wrong interpretation of the facts. Even in Fig. 2 of the CC paper one can observe that the residuals show a systematic bias when a 102 a half-life is fitted to the data. The evidence can be made more compelling by means of the simplified decay curves of both sources in Figs. 2 and 3, in which groups of 20 data points have been averaged, i.e. showing for each group the average date, average activity and its combined standard uncertainty (0.12 %/√20 = 0.027 % for source 2). Whereas the 123 a fits the data well and shows random residuals, the 102 a decay curve is clearly no match to the data and its residuals follow a monotonous upward trend. The reduced chi, *χ*_red_ = (*χ*^2^/*ν*)^1/2^, increases from 1.0 to 1.1 when replacing the fitted half-life with the erroneous value of 102 a.

It should be obvious to the objective and skilled observer that the evidence in Figs. 2 and 3 contradicts the findings of the “*careful reanalysis of the original data*” performed by CC. If the “right” result has only been obtained by mere chance, why is it the same for both sources and why have these results remained consistent throughout a measurement campaign of 2 years? The conclusion by CC that the *χ*^2^ changes “*insignificantly*” when applying 102 a instead of 120 a is invalid. The statistical quantities collected in Table 1 of the CC paper have little relevance. Their argumentation about drawing a line through the midpoint (contrary to their own practice in Fig. 3 in Ref. [2]) and the “*illustration*” of the “*meaninglessness*” (*sic*) of the fit by means of a comparison with a calibration source cannot be retained as solid.

**Table 1:** The ^209^Po alpha emission rate per unit mass *E*_α_ for SRM 4326 as a function of measurement time *t*, published by Collé et al. [2]

*t* (a)	*E*_α_ (s^−1^ g^−1^)	*S* (%)	*U* (%)
1993.21496	85.957	0.19	0.26
1993.41393	85.869	0.20	0.21
1994.18453	85.434	0.12	0.19
2005.86486	80.210	0.10	0.14
2013.92065	76.526	0.23	0.34

The quantity *S* corresponds to the relative total propagated combined standard deviation of the mean for the measurements, and *U* is the relative overall combined standard uncertainty, which includes the estimated uncertainty due to spectral interpretations and analyses

One can conclude that least squares fitting to the measurement data of both sources unambiguously demonstrate that the old value of 102 a is invalid and that publication of this conclusion in the PSB paper was justified.

### Uncertainty propagation of long-term drift

CC devised a “*warping*” function f(*t*) = (1 ± *δt*) to estimate the effect of an invisible long-term drift affecting the measurement linearly as a function of time. They freely assume a guess value of *δ* = (0.0012/359) day^−1^ for the drift in the PSB paper, which they derive from the ratio of the statistical uncertainty of one data point with the duration of the measurement campaign. Whereas these numbers should not be combined, they predict that “*positive warping*” over a period of 359 days leads to a half-life value of 152 a and “*negative warping*” to 99 a, “*which demonstrates once again that the PSB data cannot meaningfully distinguish between the half*-*lives of 102 and 120* *a*” (*sic*) [5]. However, when applying the estimated uncertainty of 0.02 % for “invisible long-term drift” in the PSB paper, CC admit that they reproduce the 3.5 % uncertainty given by PSB. One can only wonder why, at first instance, wrong data were applied to make their allegation.

Obviously, one can agree with CC’s statement that “*the validity of this uncertainty component is wholly dependent on the estimate for the possible long*-*term drift*”. Somewhat gratuitously, they ordain that “*the origin was not documented or described*” and “*more likely than not substantially underestimated*”. Of course it is an easy critique that one is unable to describe and quantify an “invisible” (and possibly non-existing!) long-term drift. In fact, it doesn’t matter what might be the origin of such long-term instability: slow geometrical changes, source deterioration, detector degradation, etc. would all have a similar effect on the half-life.

There are good reasons to believe that the measurement is performed in highly stable conditions: (1) as can been seen in Figs. 2 and 3 there are no visible medium-term trends within a level of 0.02 % over a period of 2 years and the residuals show only random variation of the magnitude as expected from Poisson statistics; (2) the sources, detectors, electronics were left untouched throughout the campaign (except for occasional power interruptions); (3) errors in dead time corrections and detector degradation are expected to be negligibly low due to the low activity of the sources; (4) the geometry of a source resting in a metal holder which fits on top of the housing of the PIPS detectors is extremely simple and its stability may be comparable to the <0.01 % geometrical reproducibility demonstrated in defined solid angle counting [11].

Note that such long-term drift, if assumed to evolve linearly in time, will remain hardly visible in the residuals of the fitted decay curve, also if the experiment is continued over a period of 20 years like in the work of Collé et al. [2]. This risk is inherent to the method. The authors see no indication that the estimated long-term drift would be larger than the estimated 0.02 %; On the contrary, this could well be a generously overestimated value. As mentioned in the conclusions of the PSB paper, confirmation can be expected when more data will become available from other decay data providers.

### Source impurity

In the PSB paper, it was mentioned that the certificate of the material provided by Eckert and Ziegler to JSI mentioned that gamma and alpha impurities were below 0.1 % at the calibration date of 27 Nov 2007. Only alpha-emissions would be detected with the same high efficiency as the ^209^Po signals. The ^208^Po and ^210^Po isotopes had been considered as possible impurity nuclides. The PSB paper included an uncertainty on the basis of a hypothetical short-lived nuclide with the half-life of ^208^Po, applying the maximum activity expected at the beginning of the experiment. CC agree that this was a reasonable assumption, but “*what of other possible impurities, including*^*210*^*Pb that would support*^*210*^*Po. No evidence was presented for any independent impurity analyses, other than an unquantified statement that no alpha peaks above 4.9* *MeV were seen in the spectrum.*” [5].

Indeed, a more detailed report on impurity studies was kept for the final paper at the end of the project. However, evidence was provided by means of a spectrum plot (cf. Fig. 1 in Ref. [4]) and the statement that no alpha peaks above 4.9 MeV were observed, which means that no significant traces of ^208^Po (5.1 MeV) and ^210^Po (5.3 MeV) were present in the material. Lead could not be present in source 1, which was prepared by self-transfer of polonium onto a silver disk. Tests at the JSI have shown that lead does not spontaneously deposit on silver, but it does deposit on stainless steel with electrodeposition, which is how source 2 was prepared.

**Fig. 4:**
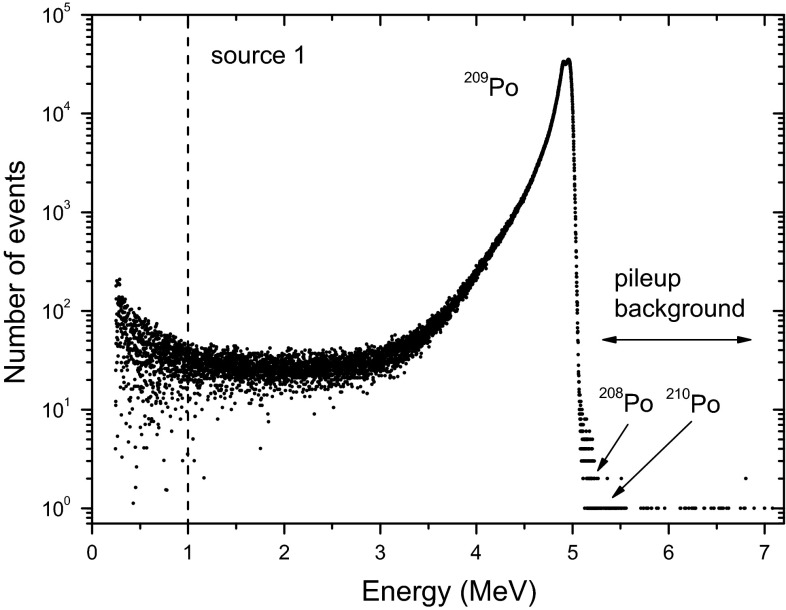
A ^209^Po spectrum of source 1 over a period of 18 days (corresponding to the ‘time gap’ in Fig. 1). The signals below 1 MeV are excluded from counting

A spectrum of source 1 taken over a period of 18 days in Fig. 4 shows 7.1 million decays of ^209^Po compared to <500 in the energy region where the impurities together with some background and pileup events are expected. A little hump after a big alpha peak due to piled-up events is sometimes observed in spectra with high counting statistics [12, 13]. No systematic temporal dependency could be observed in its relative area. Similarly, for source 2 about 300 counts were found between 5.0 and 5.3 MeV, compared to 12.4 million ^209^Po events. Therefore, the relative activity contribution from ^208,210^Po traces is certainly lower than 0.0025 %.

**Fig. 5:**
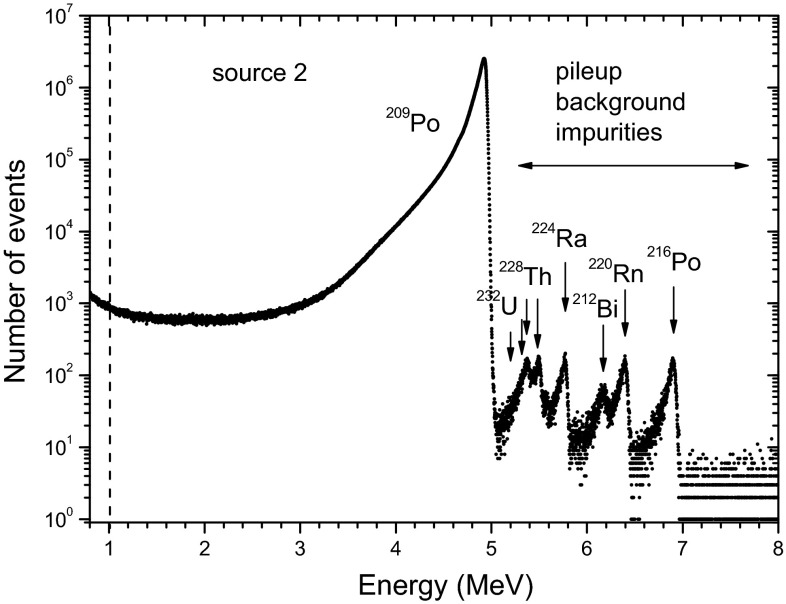
Aggregated spectrum of source 2, showing impurity from the ^232^U/^228^Th decay series

**Fig. 6:**
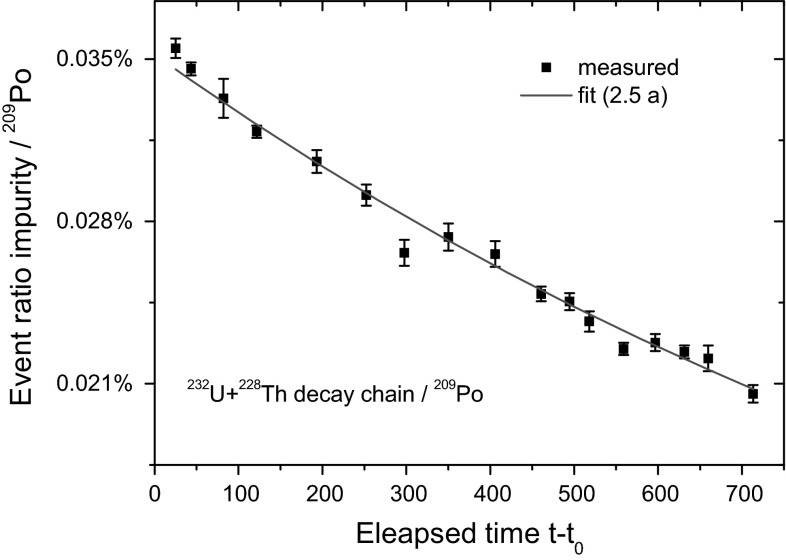
Temporal behaviour of the event ratio between ^232^U-^228^Th-daughter decays (integral 5.05–8 MeV) versus ^209^Po decays. The fitted exponential has an apparent half-life of 2.5 a

Between 5.3–8 MeV, source 1 shows no activity above background level. However, the integrated spectrum of source 2 shown in Fig. 5, reveals a set of small peaks between 5.05 and 8 MeV, consisting of 0.035 % of the count rate at the start of the campaign, but decreasing to 0.020 % after 2 years. The decay rate change corresponds to an apparent half-life of 2.5 a, as demonstrated in Fig. 6. The peaks originate from the ^228^Th (1.912 a) decay chain, partly supported by a trace of the parent ^232^U (68.9 a). Evidence of the latter could be found through the higher stability of the 5.3 MeV peak compared to the other impurity peaks. High-resolution alpha spectrometry has been performed on source 2, showing the well-separated ^209^Po peaks, but counting statistics (2 × 10^5^) was too low to observe anomalous peaks.

The impurity component in source 2 lowered the fitted ^209^Po half-life by 1.2 a, and an improved result was obtained by including the impurity fraction into the least-squares fit. A substantial uncertainty of 0.7 % was added to the half-life uncertainty budget for source 2, whereas source 1 appears to be free of impurity. In summary, one can confirm that the uncertainty estimate of 0.9 % provided in the PSB paper was realistic.

### Background subtraction

Background spectra with a dummy source were taken at the start of the measurement campaign, yielding 577 counts during 240,581 s in set-up 1 and 331 counts during 166,359 s in set-up 2. The background count rate in the detectors is low: 0.0024 (1) s^−1^ and 0.0020 (2) s^−1^ compared to the count rates of 4.6 s^−1^ and 8.0 s^−1^ produced by the sources 1 and 2, respectively.

**Fig. 7:**
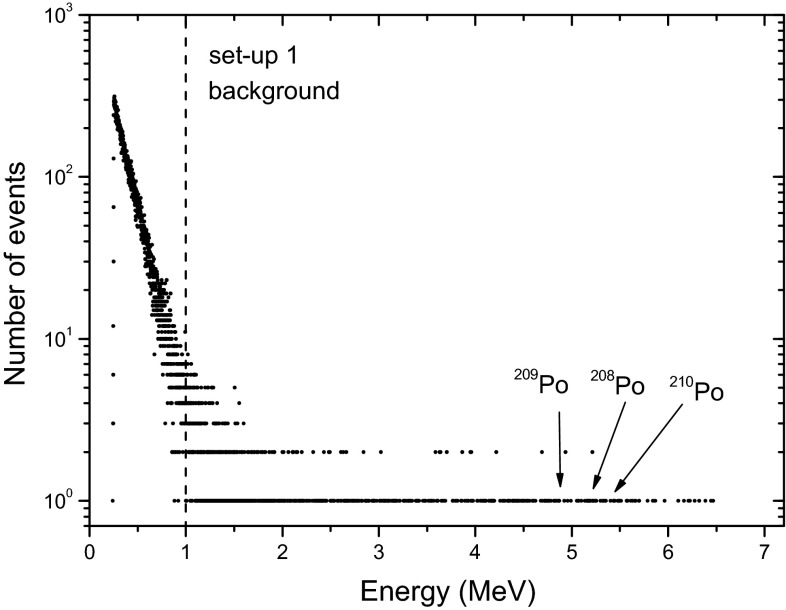
Background spectrum measured for 10 days in set-up 1, after 2 years of measurement of the ^209^Po source 1. No contamination by ^209^Po is observed

During a period of nearly 2 years, the sources were left untouched in the detectors for stability reasons and the background could not be verified. After this period, new background measurements were performed, yielding 1978 events in 887,580 s in set-up 1 and 1393 events in 887,852 s in set-up 2. The aggregate background spectrum of set-up 1 is presented in Fig. 7. In a total of 20 spectra, no variation was observed exceeding Poisson uncertainty. The count rate in set-up 1—0.00223 (5) s^−1^—has not changed significantly compared to the year 2013. The background rate in set-up 2—0.00157 (5) s^−1^—has seemingly decreased by about 21 % (which is 3.6 times the uncertainty on the difference).

In the PSB paper, a 20 % systematic error on background subtraction was taken into account, which covers this artefact. Regrettably, CC expressed suspicions about this uncertainty component (“*it is likely that they considered the 20* % *effect as a random variation in the data*” [5]), even though it was clearly mentioned as an assumed systematic error in the PSB paper. If treated as a purely random source of uncertainty, the background subtraction would be negligible in the uncertainty propagation towards the half-life. The facts today show the validity of the assumptions made in the uncertainty budget of the PSB paper.

### Source integrity

The authors agree with CC that the uncertainty about the source integrity is of real concern throughout the measurement campaign. The PSB paper mentions observations made in an early phase of the project, in which contamination of detectors were observed due to material loss from the sources. The problem disappeared after covering the sources with two VYNS foils. Since these findings pertained to tests performed in other set-ups—i.e. defined solid angle counters [14] with a geometrical efficiency of about 5 %—it was decided that a detailed description of these tests did not fit into the format of a short technical note. Measurement results obtained with these instruments will be added to the experimental evidence at the end of the project.

Firstly, the expected effect of a residual loss of material is considered: the activity of the source would decrease faster than purely through decay and the apparent half-life would be shorter than the real half-life. In the measurement set-up, part of the released material would contaminate the chamber and the detector, another part would be evacuated by the vacuum pump. The activity on the detector would be detected by a probability of about 50 %, depending on the emission angle, which would partly compensate for the loss of material. In summary, in the case of material loss the measured half-life in the PSB paper would have been lower than the real value. Obviously, this only reinforces the conclusion of the PSB paper confirming that the true half-life is significantly larger than the old reference value of 102 a.

Secondly, it has to be considered whether the obtained half-life of 123 a is an underestimate of the true half-life. After all, Collé et al. obtained a slightly higher value. For this purpose the source integrity was assessed after 2 years of continuous measurement. Visual inspection of the VYNS foils revealed no major defects, except for some small air bubbles (and a big one in source 1). The detectors were checked for possible contamination accumulated over 2 years of exposure. This was done by comparing the background spectra before and after, particularly in the region between 4.6 and 5.0 MeV, where the majority of the 4.98 MeV alpha emission events of ^209^Po are recorded.

At the start of the campaign, in the region of the ^209^Po peak there were 15 counts during 240,581 s (0.00006 s^−1^) in set-up 1 and 5 counts in 166,359 s (0.00003 s^−1^) in set-up 2. After 2 years, 38 counts in 887,580 s (0.00004 s^−1^) were recorded in set-up 1 and 13 counts in 887,852 s (0.000015 s^−1^) in set-up 2. After 2 years of ‘contaminating’ the detector, the count rate in the ^209^Po peak has not increased at all. Nor is there any sign of a concentration of background signals near 5 MeV compared to 4 or 6 MeV (see Fig. 7). Contamination by ^209^Po atoms, if any, seems to be much lower than 10^−5^ s^−1^ or less than 0.0001 % of the source count rate. Considering that the sources were facing the detector at a close distance, in nearly 2*π* configuration, it is reasonable to conclude that material loss was less than 0.001 %.

### Miscellaneous remarks

CC are so overly critical about the BSP paper that it is not opportune to counter every allegation. They dedicate half a page on “*several factual misstatements*” (*sic*) in regard to the work of Collé et al., which to their opinion is because “*the PSB authors either failed to carefully read or failed to understand the earlier work they cited*” (*sic*). The authors disagree with this point of view, since they find no valid arguments to change any of their statements.

One of the issues is a data gap, a two-week period in which it seems that no measurements were taken. This was merely caused by a human mistake after a power interruption, in which the data acquisition was accidently set to accumulate all data into one spectrum (see the spectrum shown in Fig. 4), instead of automatically refreshing the spectra on a daily basis. CC attach unnecessary mystery to this gap in the data set and even observe a change of slope “*which is a factor of 100 greater*” (*sic*) than before the gap. The reader can verify that the residuals in Fig. 1 show the same random behaviour before and after the gap.

CC seem to make a distinction between “long-term drift” and “stability of the entire measurement system”, but—as discussed in the “Uncertainty propagation of long-term drift” section—no indication of instability of source, detector, geometry could be observed and the combined uncertainty was—indeed unavoidably somewhat arbitrarily—estimated as 0.02 % over the entire measurement campaign.

According to CC “*no mention is made as to what radiations beside alpha particles were detected or deliberately excluded, such as Auger and conversion electrons and x*-*rays*”. The PSB paper did clearly mention that counts below channel 1000 (roughly corresponding to 1 MeV) were excluded from counting, which is a clear hint that all low-energy signals were eliminated.

## Comments on the *T*_1/2_(^209^Po) value by Collé et al.

### Basic analysis of the data of Collé et al.

**Fig. 8:**
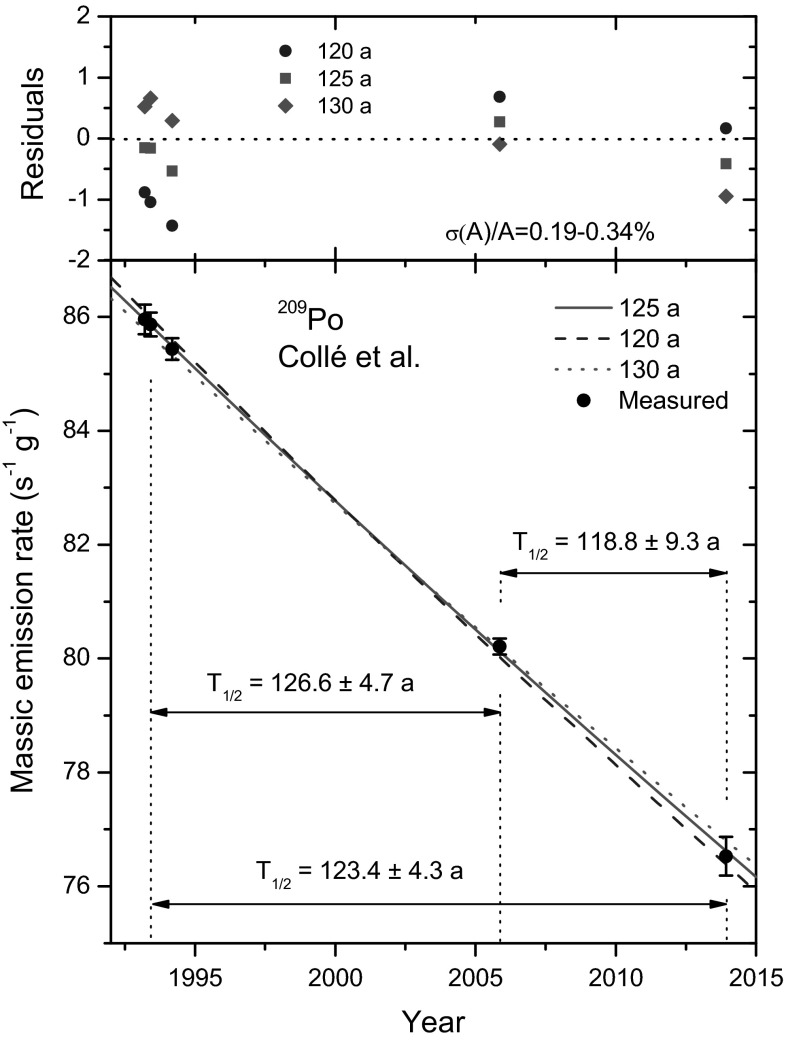
Alpha emission rates per unit mass of a ^209^Po solution measured by Collé et al. [2] and decay curves fitted through data 2, 4 and 5 using a 120, 125 and 130 a half-life (*bottom*). Residuals to the three fitted exponentials (*top*). The half-life values calculated directly from the activity ratio between data points 2, 4 and 5 are indicated in the *bottom graph*

In Table 1 and Fig. 8, the five massic alpha emission rate data measured by Collé et al. have been reproduced. The first three data are closely spaced in time and may be considered as a correlated set. One could represent this group by a mean value, or preferably by only one representative point and its uncertainty, to avoid reducing systematic errors. For the sake of simplicity, data points 2, 4 and 5 are considered as independent measurements which are linearly proportional to the activity concentration of the ^209^Po solution.

The half-life can be calculated directly from the activity ratio *A*_1_/*A*_2_ (equivalent to the measured alpha emission ratio) between two data points separated in time by an amount *T* = *t*_2_−*t*_1_, through

1 $$T_{1/2}\,=\,\frac{T\ln (2)}{{\ln (A_{1} /A_{2} )}}$$

and its uncertainty propagates through

2 $$\frac{{u_{{T_{1/2} }} }}{{T_{1/2} }} = \frac{1}{\lambda T}\sqrt {\frac{{u_{{A_{1} }}^{2} }}{{A_{1}^{2} }} + \frac{{u_{{A_{2} }}^{2} }}{{A_{2}^{2} }}} .$$

The propagation factor is 1/*λT* = 8.6 for a time period of *T* = 20.7 a. Uncertainties of 0.25 % in the activities propagate to 3 % in the ^209^Po half-life. Applying these equations to the data by Collé et al. in Table 1, one finds the following half-life estimates between data points 2, 4 and 5: *T*_1/2_(2→4) = 126.6 (47) a, *T*_1/2_(4→5) = 118.8 (93) a and *T*_1/2_(2→5) = 123.4 (43) a.

There is no exact match between *T*_1/2_(2→4) = 126.6 (47) a and the first estimate of *T*_1/2_ = 128.3 (7) a published by Collé et al. [1], since the latter appears to be derived from the 3→4 data measured 11.7 a apart [5]. The slope between the last data points 4→5 is significantly higher, leading to a shorter half-life. Ultimately, the two outmost data points lead to *T*_1/2_(2→4) = 123.4 (43) a, which is lower than the final value of *T*_1/2_ = 125.2 (33) a proposed by Collé et al. [2] and has a higher uncertainty.

### Least-squares fit of the data of Collé et al

In Fig. 8, three decay curves fitted to the data points 2,4,5 of Collé et al. [2] are shown, in which fixed values of 120, 125 and 130 a were applied for the ^209^Po half-life. As can be seen in the residuals, for all three cases a result is obtained which is statistically acceptable: the residuals of data points 2, 4, 5 remain within one standard deviation of the fitted decay curves. Collé et al. [2] derived from their least-square fits an uncertainty as low as 2.3 a, whereas the data seem to allow a variation of *T*_1/2_ by ±5 a. This is also suggested by the basic analysis in previous section, which resulted in 4.3 a uncertainty between the outer data points.

The errors were treated as purely random, whereas medium-term correlations cannot be excluded. Moreover, the total uncertainty should be large enough to anticipate for “invisible” long-term instability effects. The reader is reminded that such uncertainty components need to be propagated separately [8–10], since a least-squares fit treats them as random uncertainties and therefore erroneously reduces their propagation factor to the half-life by approximately the inverse square root of the number of fitted data. In fairness, one may assume that the systematic uncertainties were correctly taken into account by Collé et al. through the combined standard uncertainties *U* in Table 1, as well as in the propagated uncertainty component of 2.4 a for long-term effects [2].

The least-squares fit to data points 2, 4, 5 results in a 126 a half-life, because it is mainly dominated by the data points 2→4, due to the low uncertainty assigned to value 4. Arguably, *T*_1/2_(2→5) = 123.4 (43) a may be a more realistic final estimate.

### Stability and transparency issues

Stable, unchanged conditions are key to a robust half-life measurement. The standardisation measurements by Collé et al. were not designed as a half-life experiment, which explains why their half-life uncertainty over a 20.7 a measurement period is in fact larger than the one achieved in this work over a period of 2 years. The propagation factor may be 10 times smaller, but the uncertainties on the activities are an order of magnitude larger (compared to the grouped data in this work) and only three independent values have been obtained. In spite of “*the rigor employed to ensure that the measurement method and data analyses used the identical protocols in all cases*” [5], there is too much variability in the measurement conditions to guarantee optimum repeatability and transparency.

Due to lack of measurement data, there is little information about medium-term instabilities. Some elements of concern are the (in)stability of the polonium solution over this long period, the variations caused by sampling and weighing of new sources from this solution, the mixed conditions due to changes in liquid scintillation counters, scintillation cocktails, quenching, number and duration of measurements, untraceable changes in instrument settings, etc. Even with extensive reporting, the experimental design is too complex to achieve full transparency in all sources of variation.

The ensuing low reproducibility has been well illustrated by CC, who confirm that a half-life value derived from the Collé et al. measurements made over a period of 276 days (with 13 results obtained in March 2013 and 18 in November–December 2013) led to a standard uncertainty of 21 a on the fitted half-life. Having in mind the low precision achievable with these data, CC drew the wrong conclusion that the same should apply to the PSB data. Presumably, they were oblivious of the difference in repeatability achieved in both experiments.

## Finding the best value

### Updated value in this work

Whereas the PSB paper reported an intermediate result obtained over 1 year, the experiment has been pursued now for 2 years. Improvement has been achieved on statistical accuracy, the uncertainty propagation factors of long-term effects have halved and explicit checks have been performed of the background rate, source integrity (visual inspection and absence of detector contamination) and purity of the ^209^Po material. All indications show that none of the uncertainty components in the PSB paper have been underestimated. At present date, most uncertainties can be lowered significantly. Therefore, an updated half-life value and uncertainty is presented in this work.

Of source 1, 690 decay measurements with a standard deviation of 0.16 % were made over a period of 724 days. Source 2 was measured 677 times in 714 days, with a standard deviation of 0.12 % per data point. The fitted half-lives were (124.2 ± 2.9) a for source 1 and (121.9 ± 2.1) a for source 2. In the case of source 2, 0.7 % uncertainty due to the presence of impurity was added to the 1.4 % statistical uncertainty. The weighted mean is (122.7 ± 1.7) a with 1.2 % uncertainty from Poisson statistics and 0.5 % from impurity.

The spectral shape and in particular the tailing fraction remained unchanged, which is an indication that no counts were lost due to diffusion of polonium into the source backing. The count rates were corrected for dead time (<0.016 %) using a live-time technique. The uncertainty due to errors in dead-time correction, background subtraction, and material loss is insignificantly low. The only remaining uncertainty pertains to ‘invisible long-term instabilities’, for example due to slow changes in the geometrical configuration, deterioration of the detectors, drift in the electronics, source degradation, polonium diffusion, etc.

In Table 2, an overview of the uncertainty budget is presented. The uncertainty propagation factor for the Poisson statistical uncertainty was 11.7. It was calculated from [10, 15]

3 $$\frac{{\sigma_{{T_{1/2} }} }}{{T_{1/2} }} \approx \frac{2}{\lambda T}\sqrt {\frac{3(n - 1)}{n(n + 1)}} \frac{{\sigma_{A} }}{A}$$

in which *n* represents the number of data points and *σ*(*A*)/*A* the quasi-identical relative uncertainty of the uncorrelated data points, distributed evenly over the time interval [0,*T*]. A generously estimated long-term instability of 0.02 % on the activity ratio was maintained, even though there is no indication of medium-term variability of this magnitude in the residuals in Figs. 2 and 3. Due to the high propagation factor of 88 for long-term instabilities, this virtual error represents the dominant uncertainty component of 1.8 % on the half-life.

**Table 2:** The uncertainty budget for the ^209^Po half-life value obtained after 2 years of measurement in this work

Uncertainty component	*u*(*A*)/*A* (%)	*u*(*T*_1/2_)/*T*_1/2_ (%)	*u*(*T*_1/2_) (a)
Counting statistics	0.16–0.12	2.1–1.4	1.4
Activity from impurity	0.00025–0.035	0.05–0.7	0.6
Background subtraction	0.005	0.005	0.006
Dead time correction	0.00001	0.001	0.0013
Source material loss	0.0005	0.04	0.05
Hypothetical instability, Incl. geometry, electronics, Source, detector, data Analysis	0.02	1.8	2.1
Half-life	122.7 a	2.2 %	2.7 a

The propagation factor between activity ratio and half-life was 11.7 for the statistical uncertainty of 677–690 data points, and 88 for the long-term uncertainty components. The potential effect of impurities and background were calculated from theoretical considerations [10]. The final half-life value was calculated from the weighted mean for both sources using the respective combined statistical and impurity uncertainties as weighting factors. Subsequently, the systematic uncertainty components were added to the uncertainty of the mean

### A recommended value

The intermediate ^209^Po half-life result obtained after 2 years of measurement is (122.7 ± 2.7) a. This value can be combined with the result of Collé et al., of which we retain the—arguably more realistic—value derived from the basic analysis of the two outer data points: (123.4 ± 4.3) a. The weighted mean value is (122.9 ± 2.3) a, which is currently our best estimate of the ^209^Po half-life.

## Conclusions

The critique by Collé and Collé on the intermediate result of the ^209^Po half-life measurement by Pommé et al. has been refuted on every account. The published value, uncertainty and conclusion by Pommé et al. were proven to be correct in every detail. Moreover, due to a continuation of the experiment, a more precise intermediate value for the ^209^Po half-life could be presented in this work. Questions were raised about the value and uncertainty obtained by Collé et al. and an alternative half-life estimate was derived from their data.
